# A Feasibility RCT Evaluating a Play-Informed, Caregiver-Implemented, Home-Based Intervention to Improve the Play of Children Who Are HIV Positive

**DOI:** 10.1155/2018/3652529

**Published:** 2018-12-19

**Authors:** Elelwani Ramugondo, Anande Ferreira, Donna Chung, Reinie Cordier

**Affiliations:** ^1^Department of Health and Rehabilitation Sciences, Faculty of Health Sciences, University of Cape Town, Cape Town 7935, South Africa; ^2^School of Occupational Therapy, Social Work and Speech Pathology, Faculty of Health Sciences, Curtin University, Perth 6012, Australia

## Abstract

**Background/aim:**

In South Africa, contextual factors have been identified as barriers to outdoor, unstructured play. The human immunodeficiency virus (HIV) and resulting progressive HIV encephalopathy (PHE) is a pandemic in this area, associated with development delays that are not addressed by highly active antiretroviral treatment (HAART). This study aimed to describe the playfulness in children with HIV and PHE on HAART living in challenging socioeconomic areas in South Africa aged 6 months to 8 years and to evaluate the feasibility and preliminary effectiveness of a play-informed, caregiver-implemented, home-based intervention (PICIHBI) for improving play.

**Methods:**

A feasibility randomized control trial allowed for comparison of PICIHBI and conventional one-on-one occupational therapy interventions. Children were filmed playing pre-, mid-, and postintervention, using the Test of Playfulness (ToP) to assess playfulness. The PICIHBI comprised of 10 monthly sessions facilitated by an occupational therapist, involving group discussions with caregivers and periods of experiential play.

**Results:**

Twenty-four children with HIV and/or PHE were randomized into one of the two intervention groups. Overall, the group (*n* = 24) had a median score of 0 (lowest item score) on nine of 24 ToP items and only had a median score of 3 (highest score) on two items. Pre- to postintervention overall ToP scores improved marginally for the PICIHBI group (*n* = 12) and the conventional group (*n* = 12). Between-group differences were not significant. The PICIHBI group demonstrated a significant increase in one ToP item score at midassessment. No significant ToP item changes were found in the conventional group.

**Conclusion:**

Children with HIV were found to have the most difficulty on ToP items relating to the play elements of internal control and freedom from constraints of reality. The PICIHBI did not significantly improve children's play and was not more effective than the conventional intervention. Considerations for feasibility and effectiveness, including barriers to attendance, are discussed.

## 1. Introduction

Play is a primary childhood occupation and encompasses all aspects of a child's early years and development [1, 2]. Although play is not easily defined, overlapping perspectives have contributed to a widely accepted definition of play as a transaction between the individual and environment that is internally controlled, intrinsically motivated, and free from external constraints of reality and is framed by the play transaction [3–5]. The terms *play* and *playfulness* are occasionally used interchangeably; however, in this study, *play* refers to the transaction between the individual and environment and *playfulness* refers to an individual's tendency to engage in play. All play experiences are unique, as they do not involve the same set of play things, mates, or spaces, and a child brings their own interests, playfulness, and developmental skills to the play transaction. The environment is an important part of the transaction, as it can facilitate or inhibit play. Play and playfulness also occur in varying familial, cultural, social, and political contexts all of which impact on the extent to which children can be playful. Children's development is influenced by these contexts, so it is important to examine how play occurs in differing contexts and what interventions can optimise opportunities where environmental circumstances do not hinder children's play-related development.

In short, play is important for children as the context in which children develop physical, cognitive, emotional, and social skills. To play successfully, a child's own playfulness and developmental skills need to match the play task and environmental demands. This means that play is partially dependent on the achievement of certain developmental skills (e.g., gross motor skills) that a child may use to play [1]. A persistent mismatch between a child's developmental skill level, playfulness, and environment may lead to withdrawal from play opportunities. Deprivation of play can have a negative impact on a child's development [1], and playfulness has been positively linked to overall well-being [6]. Thus, occupational therapists may focus on either play as a means (using play to enhance developmental skills), play as an end (to enhance playfulness characteristics), or both, to assist children to become successful players.

For children infected with the human immunodeficiency virus (HIV), the negative impact of HIV on children's developmental milestones and areas of development needed to play (i.e., social and emotional development, behaviour, and motor and cognitive skills) are well documented [7, 8]. However, there is limited research on the play skills and aspects of playfulness which are most impacted for this population, as well as contextual factors that may contribute to lower levels of playfulness. Such information is essential in order to develop interventions that focus on using play as a means or an end to assist children in developing play and other developmental skills for this population.

HIV commonly invades the central nervous system (CNS) by means of vertical transmission. Replication of the virus within the CNS causes injury within the developing brain, leading to progressive HIV encephalopathy (PHE), associated with developmental delays [9–11]. An updated systematic review has found delays in various domains of development and executive functioning in children with HIV [8]. To date, there are no published results regarding the play patterns or development of playfulness amongst children with HIV.

In South Africa, HIV is a pandemic, with 7 million of the 55.9 million (12.5%) population estimated to be HIV positive [12]. Whilst increased use of antiretrovirals has extended people's lives, for many children, the extended family members are the primary carers of children whose parents have died of HIV-related causes. It is estimated in South Africa that there are 2.1 million children who are orphaned as a result of HIV-related parental deaths and 240,000 children are infected with HIV [13]. Therefore, many families in South Africa are affected by HIV in relation to caring arrangements, household income, and parenting and capacity.

Many children with PHE have been placed on highly active antiretroviral treatment (HAART), significantly reducing mortality and frequency of hospital admissions, allowing children to spend more time in play activities [8, 9]. However, the functioning and overall development of children within the South African context is impacted by concomitant illnesses and low socioeconomic circumstances [10, 11, 14]. For instance, low-income families may not deem play as a priority, due to concerns about daily survival needs [15]. Parental descriptions of play often include the use of toys and materials which they may not be able to afford, rather than using themselves as an active participant in play [15]. The latter is often influenced by a caregiver's working hours, which may reduce their availability to spend time engaging in play.

Another contextual factor relevant to South African children may be parental concerns about safety, which can contribute to limited opportunities for unstructured play outdoors [16]. The impact of xenophobia was found to further contribute to safety concerns and a shift from outdoor to indoor play in the Caribbean [17]. Conversely, South African children from a small community with low socioeconomic circumstances were found to engage in gross motor play, possibly because physical games do not often require equipment or materials [18]. These physical games can often have a social focus; therefore, children who are less skilful can be excluded, which can have a negative impact on playfulness.

Ferguson and Jelsma [14] and Whitehead et al. [10] highlight the need for early childhood intervention programs that address developmental concerns related to children with HIV from low socioeconomic circumstances. Occupational therapy interventions aiming to improve playfulness levels in these children must consider the contextual barriers to participation, elements of playfulness, and the manner in which the tools and knowledge are transferred to caregivers. Further, group-based interventions are important to consider where resources are limited as they increase the reach of an intervention.

This study addresses the need for early childhood intervention programs that address developmental concerns by aiming to investigate the play challenges of children with HIV and evaluating a newly developed play-informed, caregiver-implemented, home-based intervention (PICIHBI) for improving children's playfulness. This study forms part of a larger project investigating the effects of the PICIHBI on a range of developmental skills.

A feasibility randomized controlled trial (RCT) was used for this study in order to establish the viability of using the PICIHBI for improving the playfulness of children with HIV and PHE in South Africa. The aims of this study were therefore twofold and the study contained two components. The first aim and component of this study was to investigate the playfulness of children with HIV and PHE in South Africa. This first step was integral to testing the feasibility of the PICIHBI with this population as the PICIHBI gives careful consideration to playfulness elements and is intended to impart caregivers and children with strategies to alter play engagement within their homes. The second aim and component of the study was to evaluate the feasibility of a play-informed, caregiver-implemented, home-based intervention (PICIHBI) for improving children's playfulness, compared to conventional one-on-one occupational therapy.

## 2. Materials and Methods

The CONSORT statement guided the reporting of this feasibility RCT that used an assessor-blinded, block-randomized, parallel-group, pretest-posttest control-group design [19]. A feasibility RCT was used to further establish the nature of the playfulness difficulties of children with HIV, which was needed to evaluate the viability of the PICIHBI in addressing these play difficulties compared to conventional one-on-one occupational therapy. This study was needed prior to further adapting and definitively testing the preliminary effectiveness of the PICIHBI across a range of developmental skills in a larger multisite trial. Ethical approval was obtained from the University of Cape Town Human Research Ethics Committee for the larger study (HREC/REF: 560/2013) and this nested research project (HREC/REF:771/2014). The larger study is registered with the South African National Clinical Trial Registry through the National Health Research Ethics Council (trial number: DOH-27-0115-4892).

### 2.1. Participants

All children with HIV on HAART aged between 6 months and 8 years who attended the G26 out-patient paediatric antiretroviral (ARV) clinic at Groote Schuur Hospital on a monthly basis were eligible to participate in the larger study. The clinic database contained records for 142 children who met this criterion. Participants were all from low socioeconomic areas.

Attempts were made to contact families of the 142 children meeting the 6 months to 8 years age requirements contained within the hospital database. Translators assisted where needed. There were 76 children in the database who did not participate due to the following reasons: they had been transferred to a different clinic, they had been hospitalised, they were in foster care or did not have a legal guardian, they were unreachable, or they had not started HAART treatment at the time of recruitment. A total of 66 participants were randomized into the PICIHBI or conventional occupational therapy intervention groups.

After the intervention had concluded, data was available from 26 PICIHBI group participants, as four were lost to follow-up or did not attend a baseline assessment and three caregivers did not agree for their child to be filmed. Two participants in the conventional occupational therapy group were lost to follow-up or did not attend a baseline assessment and a further two caregivers did not agree for their child to be filmed, leaving a total of 29 conventional occupational therapy group participants with available data after the intervention period.

**Figure 1:**
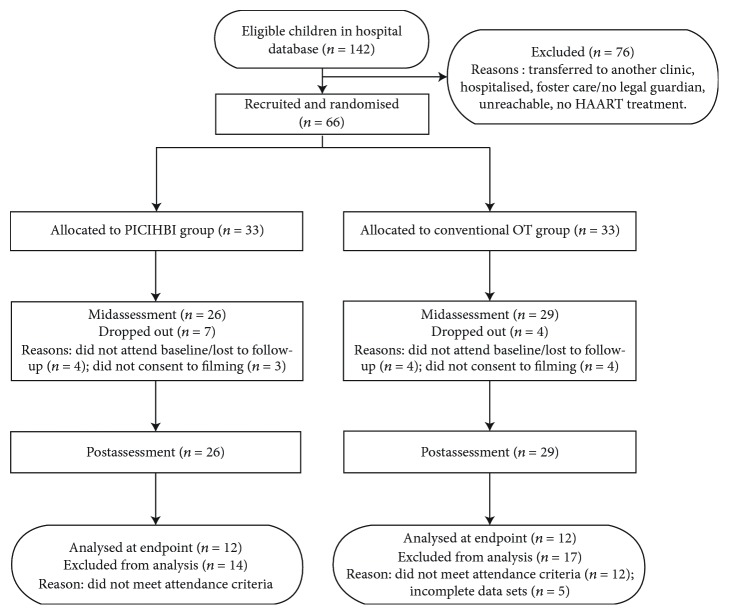
Participant flowchart.

Only data from participants who attended more than 50% of the intervention sessions were included in the analysis, as it was thought that these participants would have engaged in a majority of sessions. Children with missing playfulness data from any assessment time point were also excluded upon completion of all three assessment blocks. All data sets in the PICIHBI group were complete; however, 14 participants were excluded when the minimum attendance criterion was applied. There were 5 incomplete data sets in the conventional occupational therapy group, and 17 participants did not attend the minimum 5 sessions. The final sample consisted of 24 participants, 12 participants in each intervention group. See Figure 1 for a participant flow chart.

### 2.2. Sample Size and Power

Sample size was calculated with GPower 3.1. A total sample of 32 participants, with 16 participants per group, was required to detect the difference between the means of the two groups at a power level of 80% and a significance level of 0.05. To allow for loss to follow-up and poor attendance, the recruited 66 participants were included as part of the initial sample.

### 2.3. Randomization

A researcher independent of the project completed the randomization process for the larger research project. Therefore, participants were first randomized into two blocks stratified by age (6 months–6 years and 11 months, and 7 years–8 years and 11 months) using *Research Randomizer* [20]. Randomization to treatment groups for this study was completed within each block using *Random Sequence Generator* [21]. This resulted in two intervention and two alternative treatment groups, stratified by age. As the age stratification was required for other studies in the larger project, and not this study, the groups were collapsed to create one intervention and one alternative treatment group for this study. Randomization occurred after participants had consented to participate, and participants were blinded to the allocation process. The researchers and assessors were blinded to the allocation sequence. There were two children in the sample who were siblings, so they were randomized to the same group (PICIHBI) to avoid contamination of the intervention.

### 2.4. Instruments

#### 2.4.1. Background Information Questionnaire

A demographic and background information questionnaire was completed by caregivers. Information was collected regarding caregiver and child sociodemographics, medical and surgical history, developmental history, HAART treatment regimen and history, rehabilitative service history, schooling history, play engagement, and television habits. Caregivers were asked how they viewed their child's development, learning, and play skills in relation to their peers. This information was utilised to gain a sense of sample characteristics and an understanding of play engagement that may influence implementation of playfulness principles from the PICIHBI within the home.

#### 2.4.2. Test of Playfulness (ToP)

The tool used to measure children's playfulness was the ToP Version 4 [4]. The 29-item observational scale was designed to assess the extent (amount of time), intensity (degree of participation), and skill (ease of performance) of play behaviours of children and adolescents aged 6 months to 18 years. ToP items are operationalised according to the definition of play across four elements: intrinsic motivation, internal control, freedom from constraints of reality, and skills related to framing (reading and responding to cues). Items are ranked on a four-point scale and rated during a fifteen-minute observation of a child's free play. A raw item score of 0 is the lowest score and a score of 3 is the highest score, with higher scores indicating greater levels of playfulness. Some items may be rated “not applicable” if the child does not have adequate opportunity to demonstrate the particular playfulness skill during the 15-minute observation. The ToP is reported as clinically valid for children who are typically developing and children with developmental delay, across genders and cultural backgrounds [4, 22]. Accurate and positive construct validity (98% of respondents and 93% of test items conformed to the Rasch model) and interrater reliability (96% of raters conformed to Rasch model) are reported [4, 23]. The ToP has a moderate test-retest reliability (0.67 at *p* < 0.01) [4]. The ToP has also been used as an outcome measure in previous intervention studies involving children cerebral palsy, ADHD, and typically developing children [24–27].

#### 2.4.3. Assessment Procedures

The background information questionnaire was provided to caregivers at baseline assessment, and children were filmed playing in the clinic's play area for 15 minutes. Children were allowed to choose other children enrolled in the study or adults (namely their caregivers) as playmates, or they could play alone. The children were familiar with each other as they attend monthly clinics together. Caregivers who acted as playmates were coached to follow the child's play to ensure children had adequate opportunity to display their own levels of playfulness. The presence or absence of playmates was replicated for each child during subsequent recordings to ensure consistency over the three assessment occasions. Videos were recorded by research assistants who were also instructed to follow the child's play. Children were filmed playing on three occasions: prior to intervention, midintervention, and postintervention.

The playroom was large enough for children to be able to engage in various types of play (e.g., solitary, parallel, or games with rules) and to initiate or join play. The same toys, play materials, and other objects were available for all children during all video recordings. The toys catered to gender differences and the broad age range. Some toys may have been unfamiliar to some children due to limited exposure, but attempts were made to ensure that toys were similar to those in the playroom that they would naturally engage with when they attended other clinic appointments. Each child was given 10 minutes to explore the toys in the room before the videotape was turned on for their 15-minute video footage that would be used for assessment data. After the child's 15-minute filmed play session, caregivers were asked if their child's play at the clinic reflected their play at home. Data were also collected on intervention session attendance rates and participant dropout rates for both intervention groups.

### 2.5. Interventions

#### 2.5.1. Experimental Group: Play-Informed, Caregiver-Implemented, Home-Based Intervention (PICIHBI)

The PICIHBI focused on the caregivers of children with HIV, aiming to equip them to playfully engage with their children and to improve children's playfulness. The PICIHBI gives careful consideration to playfulness elements and is intended to impart strategies to caregivers to alter their children's play engagement within their homes. The PICIHBI also used play to facilitate fine and gross motor development, visual perceptual development, prenumeracy and preliteracy skills, and self-care. It was developed by seven occupational therapists with experience in paediatric occupational therapy. The feasibility of the PICIHBI was guided by occupational therapists experienced in working with children who are HIV positive from low socioeconomic areas within South Africa's Western Cape. Manuals were created for each age group, including detailed outlines and activities for all sessions [7].

The PICIHBI was implemented in three groups based on participant age: 6 months to 2 years and 11 months, 3 years to 5 years and 11 months, and 6 years to 8 years and 11 months. Included in the data analysis for this study were 2, 7, and 3 participants from each group, respectively. This grouping ensured skills of focus were relevant to each developmental stage. The same occupational therapist facilitated all sessions for all groups, and a translator assisted when required. The intervention comprised of 10 monthly sessions per age group, and grading options for activities were provided to allow for individual skill differences amongst the children. While the goals of the PICIHBI were not individualised for each child, they were informed by the participant's baseline assessments and were developmentally appropriate for each subgroup.

Sessions lasted 90 minutes, completed in two 45-minute parts. The first 45 minutes consisted of group discussions with caregivers to introduce the focus skill or concept for the month. The second 45 minutes was experiential, allowing each caregiver the opportunity to attempt play activities with their child to improve the earlier introduced skill in a supported environment. The occupational therapist also modelled playful behaviour to caregivers during this time, relying on caregivers to reflect internally (i.e., integrating learning from the earlier group discussion with the playful model provided by the therapist) to become more playful themselves and to create a play milieu where the children themselves would be playful. The ToP items on which children received the lowest scores during the baseline assessment were incorporated as goals of the experiential activities so that caregivers could observe the occupational therapist model these skills.

Participants (caregivers) received a “Go Box” and an information handout at each session. The Go Box contained items relevant to play, self-care, learning, and development, and the handout contained play activity suggestions that would assist caregivers to incorporate target skills into their daily routine.

#### 2.5.2. Alternative Intervention: Conventional One-on-One Occupational Therapy Intervention

Children were seen monthly, on an individual basis, by an occupational therapist who was not involved in the PICIHBI. Two different therapists were employed at different times during the research project to conduct this intervention arm. Sessions lasted 45 minutes, targeting occupational performance components relevant to the needs and assets of each child. The therapist carried out treatment sessions according to the needs identified through the baseline assessments of the larger research study and their own clinical observations. Caregivers were not the focus of the conventional one-on-one intervention; however, information on activities for the home were given to those who requested it or when deemed necessary by the occupational therapist.

### 2.6. Blinding

The researcher who scored the videos was not involved in the implementation of the interventions, was aware of the research purpose, but had no knowledge of participants' group allocation. Videos were scored after each assessment block, so the researcher was aware of the study phase when scoring. The occupational therapists running the interventions and the participants could not be blinded to group allocation.

### 2.7. Statistical Analysis

The ToP item scores of each participant were imported into the Rasch analysis Winsteps program (version 3.70.1). Rasch analysis was then completed to convert ordinal data into interval level scores. This process produced an interval level overall ToP measure score for each participant for each measurement time point. The measure scores that are generated are a function of the performance of the children within the sample against other children in the same sample. Prior to further data analysis, goodness-of-fit statistics was performed to determine infit and outfit statistics of each participant's ToP scores. This determined the goodness of fit between the ToP item scores, rater, and participants through *t*-test and mean square statistics [26]. All data conformed to the Rasch measurement model.

Analysis for the first aim and component of this study involved developing an understanding of the playfulness of children with HIV. Descriptive statistics including median and interquartile range scores were used to analyse children's ordinal-level ToP item scores at baseline to understand which items and elements of playfulness the children experienced as relatively easy or difficult.

Analysis for the second aim and component of the study involved both the overall ToP scores (interval level data) and raw ToP item scores (ordinal data) for the PICIHBI (*n* = 12) and comparison conventional (*n* = 12) groups at each assessment point. Between-group differences on ToP scores and demographic information were analysed using SPSS (version 19). *t*-Tests were used to compare continuous variables, and chi-squared tests were used to compare categorical variables between the groups. Nonparametric statistical tests were used with a combination of Friedman repeated measures and Wilcoxon Signed Rank Tests to compare within- and between-group differences on ToP items. Significance was set at *p* < 0.05. To account for the number of calculations used in this analysis, a post hoc Dunn-Bonferroni test was applied and adjusted *p* values reported. *r*-Effect size was used to calculate the effect sizes for nonparametric data. This effect size is calculated using the formula *r* = *Z*/√24. Cohen's guidelines were utilised to interpret effect sizes as small effect ≥ 0.1, medium effect ≥ 0.3, or large effect ≥ 0.5 [28].

## 3. Results

### 3.1. Participant Demographics

There were 24 participants in the sample, including 11 males and 13 females. The mean age of the PICIHBI group was 4.0 years and 4.7 years in the conventional group. The youngest participant was aged 10 months at baseline assessment and was part of the conventional group. Of the 24 children, two were reported by caregivers to have an additional diagnosis; however, the children's files indicated at least half of the participants in each group had additional diagnoses.

All caregivers were female, and high levels of unemployment were reported across both groups. Xhosa was the language spoken at home by a majority of participants in both groups. Further information on caregiver and child demographics, including caregiver perceptions of their child's play, development, and learning, is provided in Table 1.

### 3.2. Intervention Attendance

Power dropped from 80% to 68% as 26 participants from the final sample were excluded due to withdrawal, loss to follow-up, and low attendance. Participants most commonly reported missing appointments due to lack of transport money. On average, the PICIHBI group attended 7.16 out of 10 assessments, while the conventional group participants attended 6.75.

### 3.3. Aim 1: Playfulness Profile of Children with HIV

Descriptive statistics using the median baseline ToP item scores for the total sample within this study (*n* = 24 children) demonstrated that the group had a median score of 0 (reflecting a low score/skill) for nine of the 24 ToP items where sufficient data were collected. These items were *mischief* (*E*), *pretends* (*E*), *unconventional use of objects* (*E*), *clowns* (*E*), *modifies* (*S*), *supports* (*S*), *initiates* (*S*), *giving cues* (*S*), and *responding to cues* (*S*). The group had a median score of 3 (reflecting a high score/skill) for two items *decides* (*E*) and *safety* (*E*) and a median score of 2.5 for the item *engaged* (*E*). Seven items did not have sufficient data to analyse. See Table 2 for all group median baseline ToP item scores and ToP item descriptions.

Mapping these items against the element of play/playfulness each item is associated with (i.e., intrinsic motivation, internal control, freedom from constraints of reality, or framing) [23], children were found to have the least difficulty with the element of intrinsic motivation. No items associated with intrinsic motivation had a median score of 0, and the item *engaged* (*E*) had a median score of 2.5.

Within the element of internal control, children were found to have least difficulty with items associated with the area of self-control. This was demonstrated with high scores on items *decides* (*E*) and *safety* (*E*). However, children demonstrated greatest difficulty within the area of shared control. This was demonstrated by low median scores of 0 on items *modifies* (*S*), *supports* (*S*), and *initiates* (*S*). In addition to the area of shared control within the element of internal control, children also had greatest difficulty in the element of freedom from constraints of reality. This was demonstrated by low median scores of 0 on items *pretends* (*E*), *unconventional use of objects* (*E*), and *clowns* (*E*). Children also demonstrated difficulty in the element of framing with low median scores of 0 for items *giving cues* (*S*) and *responding to cues* (*S*).

### 3.4. Aim 2: Preliminary Effectiveness of the PICIHBI for Improving Children's Play

#### 3.4.1. ToP Overall Measure Scores

The mean ToP overall measure score was higher at baseline assessment for the PICIHBI group at 33.6 (SD = 17.3) than the comparison conventional group at 31.5 (SD = 23.2). However, the between-group differences in mean baseline ToP scores were not statistically significant (*p* > 0.05).

The PICIHBI group ToP measure score decreased at midintervention assessment to 28.0 (SD = 24.2), however, not to a degree of statistical significance (*p* > 0.05). The PICIHBI group ToP measure score improved from 33.6 (SD = 17.3) at baseline assessment to 35.0 (SD = 17.0) at postintervention assessment. This change was not statistically significant (*p* > 0.05).

The comparison conventional group ToP measure score increased from 31.5 (SD = 23.2) at baseline to 33.4 (SD = 23.9) midintervention assessment. This change was not statistically significant (*p* > 0.05). The group ToP measure score of the conventional group did not improve significantly from baseline assessment of 31.5 (SD = 23.2) to 38.7 (SD = 9.6) at postintervention assessment (*p* > 0.05). No statistically significant between-group differences were found between the PICIHBI group and comparison conventional intervention group at any time point (*p* > 0.05).

#### 3.4.2. ToP Item Scores

Changes in ToP item scores for the PICIHBI group are presented in Table 3. Friedman's tests showed that the PICIHBI group demonstrated a significant change in scores on four of the ToP items across time: *engaged* (*E*): *χ*^2^ = 9.1, *p* = 0.01; *decides* (*E*): *χ*^2^ = 12.8, *p* = 0.00; *social play* (*E*): *χ*^2^ = 6.4, *p* = 0.04; and *transitions* (*S*): *χ*^2^ = 9.1, *p* = 0.01. Post hoc pairwise comparison tests show that the scores of the item *engaged* (*E*) decreased significantly from baseline assessment to midintervention assessment (*Z* = 9.0, *p* = 0.01). *Decides* (*E*) significantly decreased from baseline to midassessment (*Z* = 7.0, *p* = 0.01) and improved significantly from mid- to postintervention assessment (*Z* = 8.0, *p* = 0.01; returning to baseline assessment scores). *Social play* (*E*) decreased significantly from baseline to postintervention assessment (*Z* = 7.0, *p* = 0.01). *Transitions* (*S*) improved significantly from baseline to midintervention (*Z* = 9.0, *p* = 0.01; maintaining from mid- to postintervention assessment). No significant changes were found in the comparison conventional one-on-one occupational therapy group on any of the ToP items. Results for the conventional group are presented in Table 4.

Effect sizes for ToP item scores for the PICIHBI and comparison groups are presented in Table 5. For the PICIHBI group, a total number of 11 small, medium, or large effect sizes were found across the ToP items. There was a small effect size for change in *initiates* (*S*) from baseline to midintervention. There were also small effect sizes from baseline to postintervention for *process* (*E*), *initiates* (*S*), *cues* (*S*), and *responds* (*S*). Mid- to postintervention revealed a small effect size for *cues* (*S*), *cues* (*E*), and *object* (*I*); a medium effect size for *engaged* (*E*) and *process* (*E*); and a large effect size for *decides* (*E*) (see Table 5).

For the comparison group involving conventional one-on-one occupational therapy group intervention, 11 small or medium effect sizes were found across the ToP items. There were small effect sizes from baseline to midintervention for *process* (*E*) and *cues* (*S*). There were six notable effect sizes from mid- to postintervention; small effect sizes for *pretends* (*E*), *shares* (*E*), and *cues* (*S*); and medium effect sizes for *process* (*E*), *supports* (*S*), and *responds* (*S*). Mid- to postintervention revealed small effect sizes for *engaged* (*E*) and *supports* (*S*) and a medium effect size for *decides* (*E*) (see Table 5).

## 4. Discussion

This study aimed to develop a play profile of children with HIV living in low socioeconomic contexts in South Africa and to understand whether a play-informed, caregiver-implemented, home-based intervention (PICIHBI) was feasible and showed preliminary effectiveness for developing children's playfulness. A key finding from this study was that children with HIV obtained the lowest possible score on almost 40% of ToP items that had sufficient data for analysis. These low scores were particularly pronounced in items associated with the playfulness elements of internal control, freedom from constraints of reality (pretend play), and, to a lesser extent, skills related to framing.

Interestingly, the same level of difficulty was not found in ToP items reflecting intrinsic motivation. This was demonstrated by children's relatively high scores across these items, demonstrated by their ability to be actively and intensely *engaged* in play; their ability to engage in play for the *process*, rather than an external reward; and the extent of time they engaged in *social play* with another play partner. Similarly, children with HIV were found to have relatively high scores in ToP items reflecting aspects of the element internal control [29]. This was demonstrated by their ability to *decide* what game to play and maintain a level of *safety* sufficient to play and the degree to which they used *objects* in their play. A person-item map is produced during Rasch analysis to indicate the spread of item difficulty within a measure. It is therefore important to note that the Rasch modelling of ToP items indicate that items related to internal control are relatively easy items to obtain a high score on [23].

In contrast to children's relatively high scores on ToP items associated with the play element of intrinsic motivation, they had low scores on ToP items associated with the play element of framing and some ToP items related to internal control. This was demonstrated by children actively engaged in play but lacking the interpersonal skills to maintain play interactions with another, including their ability to *initiate* play and *support* the play of others and *share* toys, space, or equipment during play with another; the skill and intensity in which they engaged in *social play* with another person; and their ability to *give* and *respond to cues* (verbal and nonverbal) during a play interaction. Children were also found to have low scores on ToP items associated with the element of freedom of constraints of reality. This was demonstrated by the lack of skills demonstrated in pretend play. Difficulty in pretend play could have adverse effects on the current and future development of emotional understanding in children with HIV, as childhood developmental theory denotes that pretend play is central to the development of emotional attunement [30].

Collectively, these findings about the playfulness of children with HIV indicate that children are motivated and able to engage in play; however, they have difficulty in areas of play that are highly dependent on their ability to interact with others. This indicates that children with HIV may have difficulty in the area of empathy (including emotional attunement and perspective-taking skills) and social interaction skills. These findings are consistent with previous studies which found that children with other developmental difficulties also demonstrate low levels of playfulness in ToP items that require these skills [26, 31–36]. No studies could be located that investigated the play of children without HIV from the same communities to enable comparison.

It is important to note that a number of factors about the play of children with HIV living in low socioeconomic contexts remain unknown. There were some ToP items that had insufficient data to be included in the analysis. To address this issue, further research is needed to gain a more ecologically valid snapshot of children's play. This will likely require play observations across multiple settings, including the child's home and a familiar outdoor play space. It is also unknown how the levels of playfulness of children with HIV compare to control children from the same socioeconomic contexts, as well as the broader population of children with HIV living in South Africa. Therefore, further research is needed to determine if these findings can be replicated within a larger more representative sample and if children's play is significantly lower than control children without HIV. This is an important avenue of research to explore, as a high percentage of caregivers within this study considered their children's play skills to be on par with or better than those of their peers. Once established, this will allow for further adaptation and refinement of the PICIHBI to better address the specific play needs of children with HIV.

A second key finding from this study was that the PICIHBI did not significantly improve the play skills of children with HIV and was not significantly more effective than the comparison conventional one-on-one occupational therapy intervention. We postulate that there are likely many factors that have impacted the feasibility and therefore effectiveness of the PICIHBI. Firstly, the loss of participants through attrition and lack of attendance suggest that there are significant barriers that prohibit the majority of families from engaging in this intervention. These findings applied to both the PICIHBI and comparison intervention. The loss of power within this study may have prevented the detection of differences between groups, if there were in fact any differences to be detected. This study nonetheless demonstrated that the PICIHBI can achieve similar results to one-on-one conventional therapy which enables more efficient use of occupational therapists' time.

A major factor may have been the high levels of unemployment within both intervention groups. The difficulties associated with high unemployment and challenging socioeconomic circumstances were demonstrated within this study with participants reporting missing appointments due to lack of transport money. Furthermore, caregivers of children with HIV experience high levels of stress and require emotional support due to the impact of HIV on family life [10, 14]. Concerns related to basic survival will overshadow the need to further understand child development, and play engagement may not be deemed a priority [15]. These challenges of time and socioeconomic constraints were evident in this study, and while attempts were made to reduce the burden on caregivers by scheduling appointments on the same day as other appointments and reimbursement of transport costs, they did not prove adequate. These findings are consistent with another study based in South Africa that also reported a high attrition rate, despite similar attempts to ease the burden of clinic attendance [10]. These findings highlight the challenges of access to early childhood intervention programs for families of children with HIV living in low socioeconomic contexts. It signifies the critical importance of providing community outreach services which lessen the burden for attendance on caregivers. The use of outreach, community-based interventions may offer the best chance for early intervention and sustained attendance.

Another major factor that may have influenced participation in the study became evident in participants' demographic data, which indicated a perception amongst most caregivers that their children's play, development, and learning were at an equivalent or higher level to those of their peers. Further, caregivers did not appear well informed about their children's concomitant diagnoses. This perception and lack of understanding may have impacted caregiver “buy-in” to the interventions, likely contributing to having missed appointments. Maternal play beliefs have a profound influence on the types of play activities and the frequency of their child's play engagement [37]. In the context of this study, education of caregivers at clinic check-ups was required prior to offering intervention. A greater understanding regarding their child's medical conditions and developmental milestones may have altered caregiver perceptions of their child's overall developmental status and needs and therefore increased motivation to attend intervention sessions.

A finding of note for the design and delivery of interventions was issues around the participant's adherence to the intervention protocol. It was noted that children did not always attend the clinic for intervention sessions with the same caregiver, usually due to work commitments. This was particularly pertinent for the PICIHBI intervention which relied on the main caregiver attending all sessions. The modelling provided by the therapist during the experiential part of the sessions was critical to improving the play behaviours and playfulness of children. Future interventions should consider ways to consistently engage the same caregiver in intervention sessions or have a family-based approach, so information and skills are passed on to other family members, including extended family members, in an easy and understandable way. Again, primary caregivers may engage more easily with interventions provided closer to home through community clinics, with flexible appointment scheduling that is compatible with other work and family commitments.

Another important aspect to consider in future studies is the intervention dosage. The findings show that very few families recruited to the study received the intended dosage of both interventions studied. Increasing intervention dosage for these families is challenging against the context of aforementioned difficulties associated with environmental stressors. Behavioural learning research indicates little outcome from monthly learning sessions at the acquisition stage of learning. This could also explain the low change in playfulness scores. The exact solution to this challenge is unclear, but part of the solution should involve considering a location more convenient to the family, such as home or community settings.

The findings from this study support recommendations of the importance of providing long-term early childhood intervention programs for children with HIV living in challenging socioeconomic circumstances in South Africa [10, 14, 38, 39]. Preparing children for schooling through an early childhood play-based intervention will provide children with more opportunity to practice, learn, and develop through play [3, 23, 32]. As play is deemed the main occupation of childhood and described as a determinant of health and well-being [3], the play needs of children with HIV should be brought to the forefront in this area of research.

Consideration and attention to the specific elements of playfulness that are challenging for children with HIV could guide future interventions, and improvements in some ToP items could guide therapists to focus on certain playfulness skills, before moving to more advanced skills. A challenge for this area of research will always be the retention of the sample when it demands high levels of attendance and commitment from participants with socioeconomic challenges and who are often experiencing high levels of stress.

### 4.1. Limitations

The high levels of attrition resulted in a small sample size, thus limiting the generalisability of the results. The loss of power in the study also potentially impacted the detection of differences between the two intervention groups. Even though it is unlikely, there is a possibility that the cluster randomization of two siblings to the same group may have had an effect on the analysis in a sample of this size. Multiple comparisons were made during data analysis, and we opted to adjust the *p* values using the post hoc Dunn-Bonferroni test. Such an adjustment may be too conservative in the case of this study and may have impacted the ability to detect significant differences. Also, baseline scores were not accounted for in the nonparametric tests, and this too may have impacted on the findings.

Furthermore, language barriers impacted the manner in which the therapists were able to engage with caregivers and children during both interventions. Therapists had to rely heavily on translators to convey information to caregivers and children, leaving open the potential for critical information to be lost.

While evaluating the effectiveness of the PICIHBI in relation to traditional occupational therapy was not a primary aim of this study, future studies of effectiveness might consider including a waitlisted control group to determine whether the PICIHBI is more effective than no intervention. Also, the PICIHBI was implemented in this study by a single therapist who was familiar with the intervention approach.

Only indoor play was filmed within this study. Concerns with safety appear to limit freedom and time spent engaging in outdoor play in most urban-based South African communities. A difference in playfulness levels may have been observed if the study was extended to observe outdoor play. Attempts were made for adequate space, toys, and materials available to the children; nonetheless, it was an unnatural play environment due to the observers and researchers filming the play. While the research assistant remained at a distance, the recording process may have made some children feel uncomfortable, thus negatively impacting on their play behaviours.

## 5. Conclusions

This study described the playfulness levels of 24 children with HIV and PHE from challenging socioeconomic contexts attending a clinic within South Africa. It is the first study to establish that children with HIV on HAART from low socioeconomic areas demonstrate low levels of playfulness, indicating that these children are at risk of occupational deprivation as they do not engage in play with ease. Though whether these challenges are unique to the population of children with HIV in this study or all children from the same low socioeconomic areas is unknown. Children with HIV obtained the lowest possible score on almost 40% of ToP items, demonstrating the need for play-focused early interventions. No significant differences were found between the PICIHBI and one-on-one conventional groups at baseline, mid-, and postassessments. Considerations of factors that reduce barriers to attendance and improve caregivers' access to intervention, such as community-based service delivery, are critical and could further contribute to improved playfulness levels for children with HIV and minimise developmental disruption for children.

## Figures and Tables

**Table 1:** Demographic information for PICIHBI and comparison conventional groups.

	PICIHBI group (*n* = 12)	Comparison group (*n* = 12)
*Caregiver particulars*		
Age of caregiver (years), mean	39.7	33.3
Sex M/F	0/12	0/12
Home language		
Xhosa	66.7%	83.3%
Afrikaans	17%	0%
Shona	8%	0%
French	8%	0%
English	0%	8%
Zulu	0%	8%
Primary caregiver		
Mother	58.3%	83.3%
Grandparent	25%	8%
Aunt	17%	0%
Other	0%	8%
Caregiver education level		
Grade 5	8.3%	8.3%
Grade 6	16.7%	0%
Grade 9	8.3%	8.3%
Grade 10	8.3%	41.7%
Grade 11	16.7%	8.3%
Year 12 (matriculation)	25.0%	33.3%
1 year post school	8.3%	0%
2 years post school	8.3%	0%
Number of adults in household, mean (SD)	2.83 (1.59)	3.58 (1.75)
Number of children in household, mean (SD)	3.25 (1.71)	2.17 (1.03)
Unemployment (%)	83.3%	75%

*Child particulars*		
Age, mean	4.0	4.7
Sex M/F	4/8	7/5
Gestation > 37 weeks (%)	66.7%	58.3%
Birth weight in kg, mean (SD)	1.83 (1.03)	2.45 (1.04)
Years on HAART, mean (SD)	5.33 (2.39)	4.50 (3.03)
First line of treatment (%)	100%	100%
Tuberculosis history (%)	83.3%	50%

*Additional diagnoses*		
None	37%	50%
Spastic diplegia	9%	0%
Failure to thrive	9%	8%
Developmental delay	9%	25%
Other	27%	17%
Missing data	9%	—

*Caregiver perceptions of child's learning development and play*		
Developing far better than peers	0%	8%
Developing slightly better than peers	0%	8%
Development on par with their peers	67%	59%
Developing slightly slower than peers	25%	17%
Very concerned with child's development	8%	8%

**Table 2:** Baseline ToP raw item scores for overall group (*n* = 24 children).

ToP item^a^ and *description*	Baseline (Pre)	
	Med	IQR
Engaged (E)^IM^: *is activity engaged*	2.5	1.0
Decides (E)^IC^: *what to do*	3.0^∗^	1.0
Safety (E)^IC^: *maintains level of safety sufficient to play*	3.0^∗^	0.0
Mischief (E)^FCR^: *engages in mischief or teasing*	0.0^∗∗^	0.0
Process (E)^IM^: *engages in activity for the sheer pleasure*	2.0	2.0
Pretends (E)^FCR^: *to be someone else, to do something else*	0.0^∗∗^	1.0
Unconventional (E)^FCR^: *incorporates objects in variable ways*	0.0^∗∗^	1.0
Social play (E)^IC^: *engages in social play*	2.0	2.0
Clowns (E)^FCR^: *or jokes*	0.0^∗∗^	0.0
Cues (E)^FR^: *gives readily understandable verbal/nonverbal cues*	1.0	2.0
Engaged (I)^IM^: *is activity engaged*	2.0	2.0
Social play (I)^IC^: *engages in social play*	1.0	0.0
Affect (I)^IM^: *demonstrates positive affect during play*	1.0	1.0
Object (I)^IC^: *interacts with objects*	2.0	1.0
Engaged (S)^FR^: *is activity engaged*	1.0	1.0
Modifies (S)^IC^: *activity to maintain challenge/make fun*	0.0^∗∗^	0.0
Social play (S)^IC^: *engages in social play*	1.0	1.0
Supports (S)^IC^: *play of others*	0.0^∗∗^	0.0
Initiate (S)^IC^: *play with others*	0.0^∗∗^	1.0
Shares (S)^IC^: *toys, equipment, friends, and ideas*	1.0	1.0
Cues (S)^FR^: *gives readily understandable verbal/nonverbal cues*	0.0^∗∗^	1.0
Responds (S)^FR^: *to others' cues*	0.0^∗∗^	1.0
Objects (S)^IC^: *interacts with objects*	1.0	1.0
Transitions (S)^IC^: *from one play activity to another*	1.0	2.0

Notes: (E) = extent item (amount of time), (I) = intensity item (degree of participation), (S) = skill item (ease of performance). ^a^Some ToP items were scored “not applicable” for some children; as a result, the following ToP items did not have enough data for analysis: Enters (S), Persists (I), Engages in playful mischief or teasing (S), Pretends (S), Creative (I), Negotiates (S), and Clowns or jokes (S). IQR = interquartile range. Item is associated with the following elements of play and playfulness: ^IM^intrinsic motivation, ^IC^internal control, ^FCR^freedom from constraints of reality, ^FR^skills related to framing. ^∗^Highest item score; ^∗∗^lowest item score.

**Table 3:** Changes in PICIHBI group ToP item scores over time.

ToP item^#^	Descriptive statistics						Friedman's		Post hoc pairwise comparison					
	Baseline		Mid		Post		Baseline-mid-post		Baseline to mid		Baseline to post		Mid to post	
	Med	IQR	Med	IQR	Med	IQR	*χ * ^2^	*p*	*Z*	*p*	*Z*	*p*	*Z*	*p*
Engaged (E)	2.5	1.0	1.0	1.8	2.0	1.0	9.06	0.01^∗^	9.00	0.01^∗^	1.29	0.26	2.28	0.13
Decides (E)	3.0	1.0	1.5	2.0	3.0	1.0	12.07	<0.01^∗^	7.00	0.01^∗^	0.20	0.66	8.00	0.01^∗^
Safety (E)	3.0	0.0	3.0	0.0	3.0	0.0	2.00	0.37	1.00	0.32	—	—	1.00	0.32
Mischief (E)	0.0	0.0	0.0	0.0	0.0	0.0	2.00	0.37	1.00	0.32	1.00	0.32	—	—
Process (E)	2.0	1.0	1.0	2.5	2.5	2.0	3.80	0.15	0.00	1.00	1.60	0.25	3.60	0.06
Pretends (E)	0.0	1.0	0.0	0.8	0.0	1.5	1.83	0.40	1.29	0.26	0.20	0.66	1.00	0.32
Unconventional (E)	0.0	0.0	0.0	0.8	0.0	0.0	0.34	0.85	0.20	0.66	0.00	1.00	0.34	0.57
Social play (E)	2.0	2.0	1.0	0.0	1.0	1.0	6.42	0.04^∗^	2.78	0.10	7.00	0.01^∗^	0.00	1.00
Clowns (E)	0.0	0.0	0.0	0.0	0.0	0.0	1.00	0.61	1.00	0.32	1.00	0.32	0.00	1.00
Cues (E)	1.0	2.0	0.5	1.0	1.0	0.8	1.60	0.45	0.67	0.42	0.00	1.00	1.60	0.21
Engaged (I)	2.0	1.0	1.0	1.8	1.0	1.0	0.39	0.83	0.20	0.66	0.00	1.00	0.40	0.53
Social play (I)	1.0	1.0	1.0	1.0	1.0	2.0	0.61	0.74	0.00	1.00	0.67	0.42	0.15	0.71
Affect (I)	1.0	1.0	0.0	0.8	0.0	1.0	4.21	0.12	2.00	0.16	5.00	0.03^∗^	0.15	0.76
Object (I)	2.0	1.0	1.0	1.0	2.0	1.0	2.24	0.33	2.67	0.10	0.00	1.00	1.00	0.32
Engaged (S)	1.0	1.0	1.0	2.0	1.0	0.0	0.44	0.81	0.15	0.71	0.50	0.48	0.15	0.71
Modifies (S)	0.0	0.0	0.0	1.0	0.0	1.0	2.00	0.37	2.00	0.16	1.00	0.32	0.34	0.57
Social play (S)	1.0	1.0	1.0	1.0	1.0	1.0	1.62	0.45	0.20	0.66	0.67	0.42	1.29	0.26
Supports (S)	0.0	1.5	0.0	1.0	0.0	1.0	0.13	0.94	0.00	1.00	0.00	1.00	0.20	0.66
Initiate (S)	0.0	0.3	0.5	1.0	1.0	1.0	2.24	0.33	1.80	0.18	1.80	0.18	0.15	0.76
Shares (S)	1.0	0.0	1.0	0.0	1.0	1.0	0.37	0.84	0.20	0.66	0.20	0.66	0.20	0.66
Cues (S)	0.5	1.8	0.5	1.0	1.0	0.0	1.43	0.49	0.15	0.71	0.15	0.71	1.60	0.21
Responds (S)	0.0	2.0	1.0	0.0	1.0	0.0	2.23	0.33	0.50	0.48	2.67	0.10	0.20	0.66
Objects (S)	1.0	1.0	1.0	1.0	1.0	0.8	3.50	0.18	2.67	0.10	0.15	0.71	2.68	0.10
Transitions (S)	1.0	2.0	1.0	2.0	1.0	1.0	9.06	0.01^∗^	9.00	<0.01^∗^	0.50	0.48	2.78	0.10

Notes: ^#^Some ToP items were scored “not applicable” for some children; as a result, the following ToP items did not have enough data for analysis: Enters (S), Persists (I), Engages in playful mischief or teasing (S), Pretends (S), Creative (I), Negotiates (S), and Clowns or jokes (S); IQR = interquartile range; Friedman's two-way ANOVA; post hoc pairwise comparison tests. *p*: adjusted *p* value after post hoc Dunn-Bonferroni test; —: test did not run as the mean rank values were identical; ^∗^*p* < 0.05.

**Table 4:** Changes in conventional occupational therapy group ToP item scores over time.

ToP item^#^	Descriptive statistics						Friedman's		Post hoc pairwise comparison					
	Baseline		Mid		Post		Baseline-mid-post		Baseline to mid		Baseline to post		Mid to post	
	Med	IQR	Med	IQR	Med	IQR	*χ * ^2^	*p*	*χ * ^2^	*p*	*χ * ^2^	*p*	*χ * ^2^	*p*
Engaged (E)	2.5	1.8	2.0	1.0	2.5	1.0	0.71	0.71	0.11	0.74	0.50	0.48	0.50	0.48
Decides (E)	3.0	1.8	2.5	1.0	3.0	0.8	2.37	0.31	0.15	0.71	1.29	0.26	2.00	0.18
Safety (E)	3.0	0.0	3.0	0.0	3.0	0.0	4.00	0.14	2.00	0.16	2.00	0.18	—	—
Mischief (E)	0.0	0.0	0.0	0.0	0.0	0.0	2.00	0.37	1.00	0.32	1.00	0.32	—	—
Process (E)	1.5	2.0	2.0	1.0	2.0	1.0	1.25	0.54	0.11	0.74	1.34	0.25	0.15	0.71
Pretends (E)	0.0	1.0	0.5	2.3	0.5	1.3	1.85	0.40	1.29	0.26	0.67	0.42	1.29	0.26
Unconventional (E)	0.0	1.0	0.0	0.0	0.0	0.0	3.50	0.18	1.80	0.18	1.80	0.18	1.00	0.32
Social play (E)	2.0	2.0	1.0	2.0	1.0	1.0	2.68	0.27	2.67	0.10	1.80	0.18	0.20	0.66
Clowns (E)	0.0	0.0	0.0	0.0	0.0	0.0	1.00	0.61	0.00	1.00	1.00	0.32	1.00	0.32
Cues (E)	1.0	1.8	0.5	2.0	1.0	1.0	0.16	0.92	0.11	0.74	0.00	1.00	0.11	0.74
Engaged (I)	2.0	1.8	2.0	2.0	2.0	1.5	0.38	0.83	0.11	0.74	0.15	0.71	0.40	0.53
Social play (I)	1.0	1.0	1.0	2.0	1.0	1.0	0.30	0.86	0.15	0.71	0.34	0.57	0.00	1.00
Affect (I)	0.0	1.8	0.0	1.0	1.0	1.0	0.06	0.97	0.00	1.00	0.00	1.00	0.15	0.71
Object (I)	2.0	1.0	2.0	2.0	2.0	0.0	0.21	0.90	0.00	1.00	0.67	0.42	0.00	1.00
Engaged (S)	1.0	1.0	1.0	1.0	1.0	0.0	1.27	0.53	0.00	1.00	0.50	0.48	1.29	0.26
Modifies (S)	0.0	0.3	0.0	1.0	0.0	1.0	0.30	0.86	0.00	1.00	0.20	0.66	0.20	0.66
Social play (S)	0.0	1.0	0.0	2.0	1.0	1.0	0.30	0.86	0.15	0.71	0.34	0.57	0.00	1.00
Supports (S)	0.0	0.0	0.0	0.3	0.5	1.3	3.50	0.18	2.00	0.16	3.00	0.09	0.20	0.66
Initiate (S)	0.0	1.0	0.0	0.0	0.0	2.0	1.29	0.53	0.20	0.66	0.34	0.57	0.34	0.57
Shares (S)	0.0	1.5	1.0	0.3	1.0	1.3	2.63	0.27	1.00	0.32	1.80	0.18	1.80	0.18
Cues (S)	0.0	1.0	1.0	2.0	1.0	2.0	0.62	0.74	0.50	0.48	0.20	0.66	0.00	1.00
Responds (S)	0.5	1.0	0.0	2.0	1.0	1.8	3.00	0.23	0.67	0.42	2.67	0.10	1.29	0.26
Objects (S)	1.5	1.0	1.0	1.0	1.0	0.0	3.16	0.21	0.50	0.48	2.78	0.10	1.00	0.32
Transitions (S)	1.5	2.0	1.5	1.3	1.5	1.0	0.71	0.71	0.11	0.74	0.15	0.70	0.00	1.00

Notes: ^#^Some ToP items were scored “not applicable” for some children; as a result, the following ToP items did not have enough data for analysis: Enters (S), Persists (I), Engages in playful mischief or teasing (S), Pretends (S), Creative (I), Negotiates (S), and Clowns or jokes (S); IQR = interquartile range; Friedman's two-way ANOVA; post hoc pairwise comparison tests. *p*: adjusted *p* value after post hoc Dunn-Bonferroni test; —: test did not run as the mean rank values were identical; *p* < 0.05.

**Table 5:** Effect sizes of ToP item scores.

ToP item	PICIHBI group						Comparison group					
	Baseline to mid		Baseline to post		Mid to post		Baseline to mid		Baseline to post		Mid to post	
	*Z*	*r*	*Z*	*r*	*Z*	*r*	*Z*	*r*	*Z*	*r*	*Z*	*r*
Engaged (E)	−2.81^∗∗^	−0.57	−1.27	−0.26	1.71	0.35+	−0.12	−0.02	0.00	0.00	1.10	0.22+
Decides (E)	−2.43^∗^	−0.50	0.00	0.00	2.64^∗∗^	0.54+	−0.17	−0.04	0.00	0.00	1.61	0.33+
Safety (E)	0.00	0.00	0.00	0.00	0.00	0.00	0.00	0.00	0.00	0.00	0.00	0.00
Mischief (E)	0.00	0.00	0.00	0.00	0.00	0.00	0.00	0.00	0.00	0.00	0.00	0.00
Process (E)	−0.59	−0.12	1.12	0.23+	2.00^∗^	0.41+	0.73	0.15+	1.57	0.32+	0.00	0.00
Pretends (E)	0.00	0.00	0.00	0.00	0.00	0.00	−1.72	−0.35	1.19	0.24+	0.00	0.00
Unconventional (E)	0.00	0.00	0.00	0.00	0.00	0.00	0.00	0.00	0.00	0.00	0.00	0.00
Social play (E)	−2.13^∗^	−0.43	−2.46^∗^	−0.50	0.00	0.00	−1.73	−0.35	−1.66	−0.34	0.00	0.00
Clowns (E)	0.00	0.00	0.00	0.00	0.00	0.00	0.00	0.00	0.00	0.00	0.00	0.00
Cues (E)	−0.54	−0.11	0.00	0.00	0.92	0.19+	−0.06	−0.01	0.00	0.00	0.31	0.06
Engaged (I)	−0.97	−0.20	−0.54	−0.11	0.00	0.00	0.00	0.00	0.00	0.00	0.00	0.00
Social play (I)	0.00	0.00	0.00	0.00	0.00	0.00	0.00	0.00	0.00	0.00	0.00	0.00
Affect (I)	−1.51	−0.31	−2.24^∗^	−0.46	0.00	0.00	0.00	0.00	0.30	0.06	0.26	0.05
Object (I)	−1.08	−0.22	0.00	0.00	0.75	0.15+	0.00	0.00	0.00	0.00	0.00	0.00
Engaged (S)	0.00	0.00	0.00	0.00	0.00	0.00	0.00	0.00	0.00	0.00	0.00	0.00
Modifies (S)	0.00	0.00	0.00	0.00	0.00	0.00	0.00	0.00	0.00	0.00	0.00	0.00
Social play (S)	0.00	0.00	0.00	0.00	0.00	0.00	0.00	0.00	0.27	0.06	0.11	0.02
Supports (S)	0.00	0.00	0.00	0.00	0.00	0.00	0.00	0.00	1.63	0.33+	0.71	0.14+
Initiate (S)	0.96	0.20+	1.34	0.27+	0.00	0.00	0.00	0.00	0.00	0.00	0.00	0.00
Shares (S)	0.00	0.00	0.00	0.00	0.00	0.00	0.28	0.08	1.34	0.27^∗^	0.00	0.00
Cues (S)	0.00	0.00	0.63	0.13+	0.83	0.17+	0.58	0.12+	0.71	0.14+	0.00	0.00
Responds (S)	0.51	−0.10	1.00	0.20+	0.00	0.00	−0.97	−0.20	1.63	0.33+	0.34	0.07
Objects (S)	0.00	0.00	0.00	0.00	0.00	0.00	−0.72	−0.15	−1.35	−0.28	0.00	0.00
Transitions (S)	0.00	0.00	0.00	0.00	0.00	0.00	0.00	0.00	0.00	0.00	0.00	0.00

Notes: The *r*-effect size was used to calculate the effect sizes for nonparametric data. In this calculation, the effect size (i.e., *r*) is obtained by dividing the Wilcoxon *Z* score by the square root of the sample size; *r* = *Z*/√24. Cohen's guidelines for *r* are small effect ≥ 0.1, medium effect ≥ 0.3, or large effect ≥ 0.5; +: small, medium, and large effect sizes; ^∗^*p* < 0.05, ^∗∗^*p* < 0.01, ^∗∗∗^*p* < 0.001.

## Data Availability

The raw ToP data used to support the findings of this study are restricted by the University of Cape Town Human Research Ethics Committee in order to protect patients. Data are available from the first author for researchers who meet the criteria for access to confidential data.
